# Enhancing Secondary Metabolite Production in Actinobacteria Through Over-Expression of a Medium-Sized SARP Regulator

**DOI:** 10.3390/ijms262311723

**Published:** 2025-12-03

**Authors:** Elena Heng, Lee Ling Tan, Yi Wee Lim, Winston Koh, Siew Bee Ng, Yee Hwee Lim, Dillon W. P. Tay, Fong Tian Wong

**Affiliations:** 1 Molecular Engineering Lab, Institute of Molecular and Cell Biology (IMCB), Agency for Science, Technology and Research (A*STAR), 61 Biopolis Drive, #07-06, Proteos, Singapore 138673, Singapore; 2 Institute of Sustainability for Chemicals, Energy and Environment (ISCE^2^), Agency for Science, Technology and Research (A*STAR), 8 Biomedical Grove, #07-01, Neuros Building, Singapore 138665, Singaporelim_yee_hwee@a-star.edu.sg (Y.H.L.); 3 Bioinformatics Institute (BII), Agency for Science, Technology and Research (A*STAR), 30 Biopolis Street, #07-01, Matrix, Singapore 138671, Singapore; 4 Singapore Institute of Food and Biotechnology Innovation (SIFBI), Agency for Science, Technology and Research (A*STAR), 31 Biopolis Way, #01-02, Nanos, Singapore 138669, Singapore; 5 Synthetic Biology Translational Research Program, Yong Loo Lin School of Medicine, National University of Singapore, 10 Medical Drive, Singapore 117597, Singapore

**Keywords:** *Streptomyces*, Actinobacteria, *Streptomyces* antibiotic regulator protein, secondary metabolites, natural products

## Abstract

Natural products hold immense therapeutic potential, yet they remain underexplored due to challenges in activating or producing them in laboratory settings. Here, we investigate the regulatory capabilities of a new medium-sized *Streptomyces* Antibiotic Regulator Protein (SARP), Fzm_SARP, in comparison to the well-characterized small SARP, RedD, across 18 diverse actinobacterial strains. In addition to the conserved DNA-binding domains typical of SARP regulators, the medium-sized Fzm_SARP also contains an additional NTPase domain. Our study revealed that 327 of the 422 metabolites (77%) detected in 18 wild-type actinobacterial strains were up-regulated in the SARP over-expressing strains. Among these 422 metabolites, 55% were up-regulated in the two SARP over-expressing strains whereas 15% and 7% were specifically up-regulated in the RedD and Fzm_SARP over-expressing strains, respectively. Interestingly, 244 metabolites not previously detected in the wild-type strains were detected in the two SARP over-expressing strains, resulting in a 58% increase from 422 to 666 metabolites. 36% of these new 244 metabolites were up-regulated in the two SARP over-expressing strains whereas 37% and 27% of these metabolites were specifically up-regulated in the RedD and Fzm_SARP over-expressing strains, respectively. These regulator-specific metabolites also give rise to distinct bioactivity profiles observed for each SARP. Overall, these findings expand our understanding of SARP family regulators and offer valuable insights for future research and applications in microbial biotechnology and secondary metabolite production.

## 1. Introduction

Natural products have significant therapeutic potential yet remain underutilized despite advancements in genomics and bioinformatics [1,2]. A growing bottleneck is the low metabolic state of lab-fermented strains, which often necessitates further engineering for sufficient production. These efforts include refactoring native promoters within biosynthetic gene clusters (BGCs) [3,4], heterologous production [5,6,7,8], and optimizing cultivation [9]. However, these are still limited by the native complex regulation of secondary metabolites within these microbes [10,11]. Despite this limitation however, the interconnectivity of these regulation networks can also be exploited as a tool for up-regulation.

One strategy that had great success in our lab [12,13,14], is the use of genetic tools to manipulate regulator levels (inactivation or over-expression). This led to the unlocking of the vast biosynthetic potential of these bacteria resulting in an increase in secondary metabolite production. This approach is advantageous, compared to systems biology or pathway engineering, since it can be used across various actinobacterial species and BGCs. For instance, the over-expression of an activating regulator is particularly appealing to explore the biosynthetic abilities of a library of uncharacterized strains, especially when it is combined with the “One Strain Many Compounds” (OSMAC) approach [15,16,17].

Numerous studies dedicated to the analysis of transcriptional and translational regulatory elements in various *Streptomyces* genomes [18] are revealing intriguing insights about regulators. Regulators were previously identified by their genomic location: specific and global. However, evidence shows that specific regulators clustered within BGCs can exhibit non-specific behavior. In particular, we focus on the *Streptomyces* Antibiotic Regulator Proteins (SARPs), which are widely distributed within gene clusters in actinobacteria, especially in *Streptomyces* [19]. Despite their “specific” designation, they have been demonstrated to function beyond their specific BGCs—PapR and RedD [20]. However, these examples are mainly representatives from the group of “small SARP” regulators less than 400 amino acids long, each with a OmpR DNA-binding domain (OBD) and a Bacterial Transcriptional Activation domain (BTAD) (e.g., ActII-ORF4 or RedD). It is hypothesized that they have similar protein architecture and DNA-binding sequences that constitute the basis of their functional abilities across actinobacterial species and BGCs. However, there are less well characterized classes of SARPs, including medium and large SARPs, which contain an additional NTPase domain and/or a conserved C-terminal tetratricopeptide repeat (TPR) domain of unknown function (~600–1000 residues) [21] which are similarly abundant as the small SARPs [22].

Recently, we constructed over-expression cassettes of SARP-encoding genes that were integrated in the genome of several actinobacterial strains via phiC31 recombinase. The implementation of this strategy led to the rapid activation of several biosynthetic pathways present in strains of an actinobacteria library [14]. In this study, using this protocol, we characterized the first example of a medium SARP acting as a regulator activator for secondary metabolite production across multiple actinobacteria species.

## 2. Results

### 2.1. In Silico SARP Comparisons

**Figure 1:**
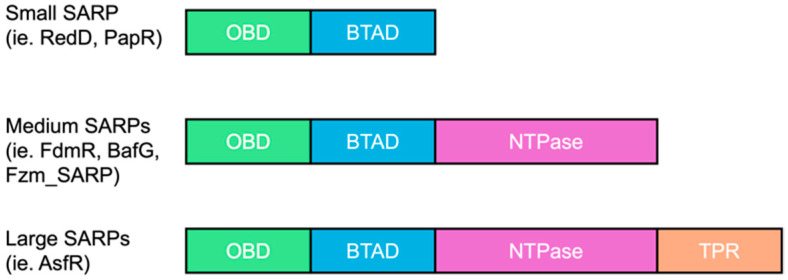
Comparison of different SARP types. Definitions: OmpR DNA-binding domain (OBD) and Bacterial Transcriptional Activation domain (BTAD), tetratricopeptide repeat (TPR).

We chose to study a medium-sized SARP (WP_078621770.1, called Fzm_SARP hereafter, Figure 1) transcriptional regulator in a fosfazinomycin A-like cluster in *Streptomyces* sp. NRRL S-244 (Table S1). Here, we observed that Fzm_SARP is 97.36% identical to AGZ93914.1 in the fosfazinomycin A biosynthetic gene cluster [23] from *Streptomyces* sp. WM6372 (miBiG BGC0000937). However, Fzm_SARP (665 amino acids long) contains OBD, BTAD, and NTPase domains while AGZ93914.1 (341 amino acid long) only contains the NTPase domain. To study further this particular medium-sized SARP, we compared the occurrence and the type of BGCs present in close vicinity of these SARPs in various actinobacteria genomes, using an extensive database of actinobacteria genomes [14,24]. Results are shown in Table 1. We noticed that although OBD and BTAD domains were conserved, these SARPs share less than 42% identity. In contrast to small SARPs primarily found in *Streptomyces* species, medium-sized SARPs are present in the BGCs of both *Streptomyces* and *Kitasatospora* species.

While the ability of small-size SARPs (PapR and RedD) to act as global regulators across various *Streptomyces* species has been previously demonstrated [20], the stimulatory effect of medium SARPs (FdmR and BafG) on specialized metabolite production has only been demonstrated in their original strains and for specific metabolites of the latter [19,25]. In consequence, our study of Fzm_SARP gives us the opportunity to determine whether this medium-sized SARP can also act as a global regulator across various *Streptomyces* species.

### 2.2. Comparison of Activation Potentials Across Actinobacteria

To assess the effectiveness of Fzm_SARP as a general activator for enhancing metabolite production in actinobacterial strains, we compared its effects on metabolite diversity and abundance with those of a prototypical small SARP, RedD, across various actinobacteria. We integrated an over-expression cassette placing Fzm_SARP under kasO*p into 18 distinct actinobacterial species. These strains mainly consist of *Streptomyces*, but also included *Thermoactinomyces* and *Micromonospora*, all of which were isolated from soil and marine environments in Singapore [27] (Table S2). Although the RedD and Fzm_SARP datasets were collected concurrently, the results for the RedD dataset have been published in an earlier study [14].

The resulting mutants over-expressing Fzm_SARP were fermented in 3–5 different media (Tables S3 and S4). Fermentation extracts were analyzed using LC-MS/MS, and the resulting spectral data processed through the Global Natural Products Social Networking (GNPS) [28] molecular networking workflow to cluster compounds with similar fragmentation patterns (Tables S5 and S6). MS intensities of individual metabolites were used to determine abundance fold changes (Table S7) due to the over-expression of Fzm_SARP compared to RedD over-expression [29,30].

**Figure 2:**
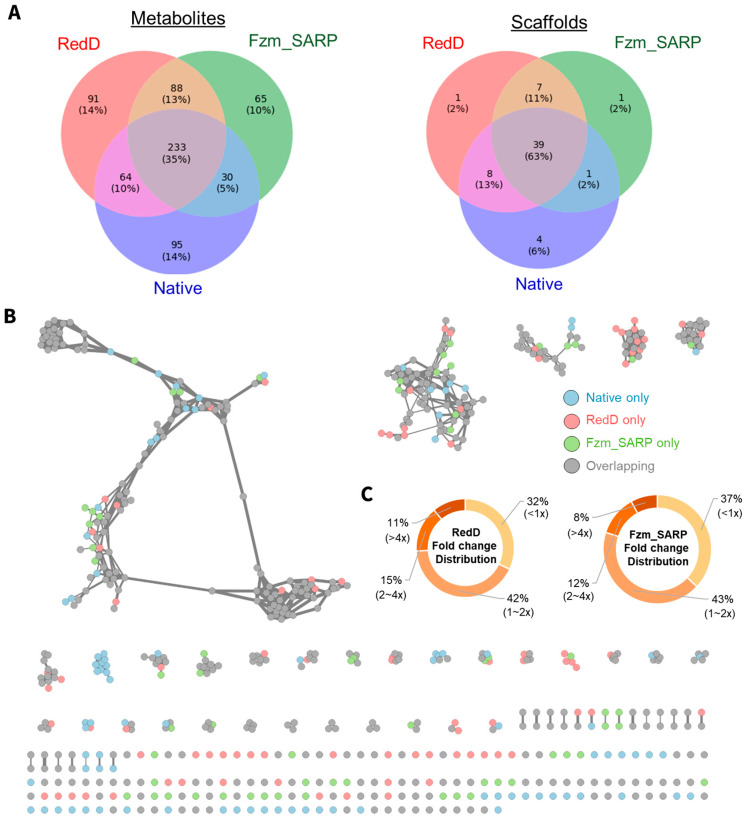
RedD and Fzm_SARP effects on metabolite diversity and abundance for representative mutants from 18 actinobacterial strains in 3–5 media. (**A**) Venn diagram showing distribution of 666 metabolites and 61 scaffolds (i.e., clusters with ≥2 metabolites) identified via GNPS [28]. (**B**) Molecular network of metabolites with metabolites unique to native (blue), RedD (red), and Fzm_SARP (green) indicated. (**C**) Fold change in metabolite abundance for those produced by both wild-type as well as mutant strains for RedD and Fzm_SARP mutants.

The comparison of metabolite coverage revealed that both RedD and Fzm_SARP activation had similar improvements in metabolite coverage, both yielding 8 new scaffolds each but 179 and 153 new metabolites, respectively (Figure 2A, sum of unique and overlapped not observed in native). The significant overlap of 7 new scaffolds and 88 new metabolites activated both by RedD and Fzm_SARP suggest that they may target similar biosynthetic pathways. Unique metabolites produced by each activation strategy are detailed in Figure 2C, highlighting both the shared and distinct contributions of these activators. To further investigate these effects, we performed strain-level fold change comparisons of shared metabolites (Figure 2B, Table S7). On average, RedD activated strains yielded higher producers (~2.4-fold average increase vs. wild-type) compared to Fzm_SARP activated strains (~2-fold average increase vs. wild-type), indicating that while both modifications have similar influence on metabolite diversity, RedD has a more pronounced impact on metabolite abundance.

### 2.3. Case Study: Surfactins and Nocardamines

**Figure 3:**
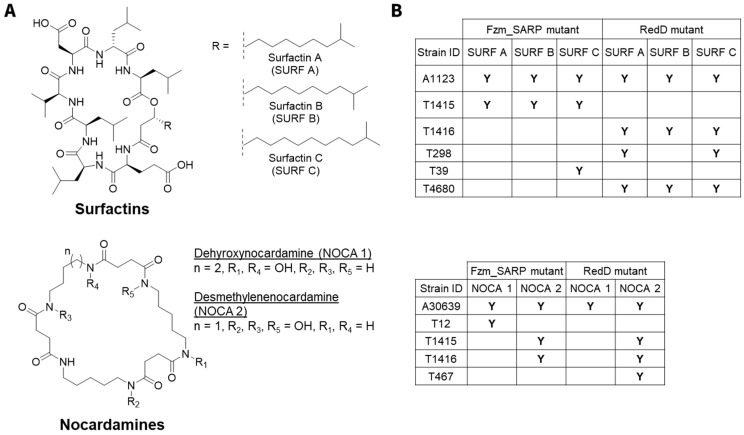
RedD and Fzm_SARP effects on surfactins and nocardamines for 18 actinobacterial strains in 3–5 media. (**A**) Molecular structures of surfactin analogues A (SURF A), B (SURF B), and C (SURF C) as well as of nocardamine analogues, dehydroxynocardamine (NOCA 1) and desmethylenenocardamine (NOCA 2). (**B**) Presence of surfactin and nocardamine analogues across mutant strains. “Y” indicates the compound is detected in the mutant strain as a result of activation and is only observed in mutant. These compounds were not observed in any of the wild-type strains.

Both Fzm_SARP and RedD exert significant activating effects on surfactin and nocardamine production across multiple strains, though with distinct patterns (Figure 3). When comparing Fzm_SARP with RedD, the smaller RedD SARP showed a more widespread activation profile, consistent with its well-documented ability to act as a cross-cluster regulator. The broader responsiveness of RedD may be linked to its streamlined domain architecture (OBD + BTAD) and conserved binding motifs, which have been shown to facilitate its regulatory flexibility [22,31].

### 2.4. Bioactivity Comparison

**Figure 4:**
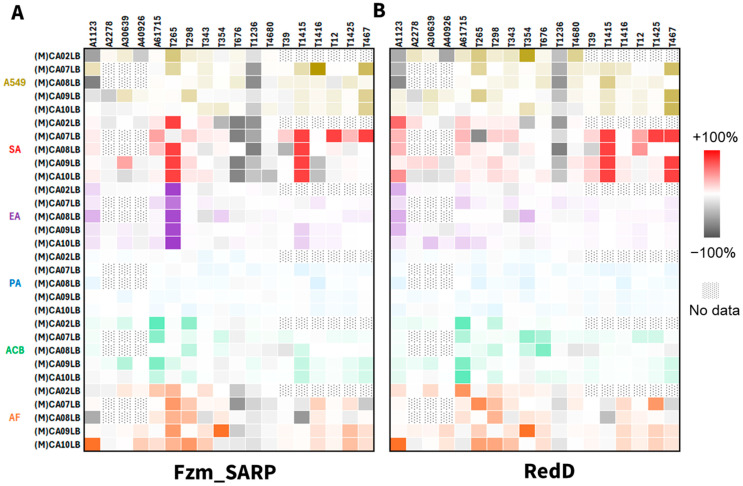
Differential bioactivity profiling of fermentation extracts of mutant versus wild-type strains from combinations of 18 actinobacterial strains (above heat map) in 3–5 media (left of heat map). Summarized for strains integrated with (**A**) Fzm_SARP, and (**B**) RedD. A549 (brown) = cell cytotoxicity against human lung carcinoma cells. SA (red) = antibacterial activity against *Staphylococcus aureus*. EA (purple) = antibacterial activity against *Klebsiella aerogenes*. PA (blue) = antibacterial activity against *Pseudomonas aeruginosa*. ACB (green) = antibacterial activity against *Acinetobacter baumannii*. AF (orange) = antifungal activity against *Aspergillus fumigatus*. Heat maps were constructed using the methods described in a prior study [14].

In addition to analyzing chemical profiles, we aimed to investigate whether these up-regulated metabolites exhibited differences in bioactivity. Fermentation extracts from the 18 wild-type strains and their RedD [29] or Fzm_SARP activated mutants were evaluated for inhibitory bioactivity against *Staphylococcus aureus* (SA), *Klebsiella aerogenes* (EA), *Pseudomonas aeruginosa* (PA), *Acinetobacter baumannii* (ACB), *Aspergillus fumigatus* (AF) and cell cytotoxicity against human lung carcinoma cells (A549). Visualizing the bioactivity of fermentation extracts from RedD mutants (Table S8) versus Fzm_SARP mutants (Figure 4, Table S9) revealed broadly similar shifts in bioactivity profiles, alongside several notable differences unique to each regulator. For example, both SARPs increased inhibition of *Staphylococcus aureus* for T1415 across all examined media.

It is noteworthy that Fzm_SARP activation maintains a distinct advantage in selected strains, indicating the presence of unique bioactive metabolites. For example, T265 shows broad improvement in inhibition against *Klebsiella aerogenes* (EA, purple) and *Staphylococcus aureus* (SA, red) due to Fzm_SARP integration but not from RedD integration (Figure 4). When we examine the up-regulation across individual strain-media combinations, the results vary, with some favoring Fzm_SARP (e.g., cell cytotoxicity against human lung carcinoma cells, A549, brown, for T1416 in MCA07LB media) and others favoring RedD (e.g., inhibition of *Staphylococcus aureus*, SA, red, for T467 in MCA09LB or MCA10LB media), highlighting the importance of different regulators.

## 3. Discussion

Our study showed that, despite low sequence similarity among SARPs, similar patterns in activating secondary metabolites were observed. Notably, a high percentage of shared scaffolds were activated in both RedD and Fzm_SARP. In silico analysis of the native Fzm gene cluster revealed binding motifs with consensus sequences similar to those of RedD and PapR (Figure 2A). Structural predictions of RedD and Fzm_SARP, followed by alignment, revealed RMSDs of 1.6 Å between RedD and AfsR, 2.0 Å between Fzm_SARP and AfsR, and 2.2 Å between the predicted RedD and Fzm_SARP models. These results indicate a high degree of structural similarity among these regulatory proteins. Despite low amino acid sequence conservation, the domains responsible for regulatory functions are highly conserved, highlighting an evolutionary strategy: maintaining essential domains while allowing expansion to enable pathway activation.

**Figure 5:**
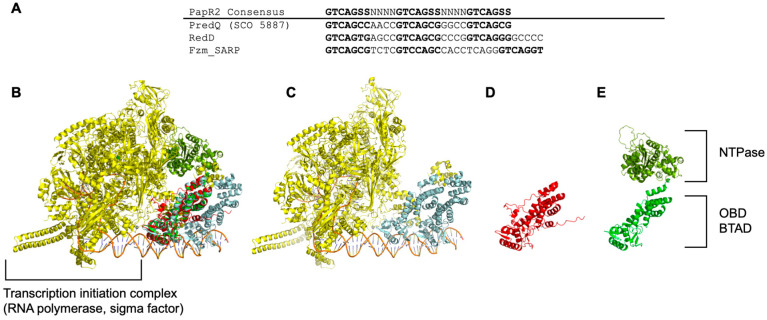
In silico analyses of Fzm_SARP. (**A**) Comparison of predicted SARP-binding sequences in the upstream regions of RedQ (SCO5887) regulated by RedD, and putative binding sites upstream of FzmF (ctg1_97) associated with Fzm_SARP (GCF_000717915.1). (**B**,**C**) Structural alignment of the predicted Fzm_SARP (green) and RedD (red) models with the cryo-EM structure of the *Streptomyces coelicolor* transcription initiation complex containing the global regulator AfsR (yellow; PDB ID: 8K60). Predicted domain organization of (**D**) RedD (red) and (**E**) Fzm_SARP showing the NTPase and OBD–BTAD. Further methodological details are provided in Section 4.

The NTPase domain found in Fzm_SARP signifies a different evolutionary advancement for medium-sized SARP compared to smaller SARPs like RedD, which lacks this domain. Although the cryo-EM structure of the large SARP, AfsR, reveals the presence of NTPase and TPR domains, only the OBD and BTAD are visible, indicating flexibility of the NTPase and TPR domains within the overall structure [22,31]. Structural alignment of Fzm_SARP with AfsR in the transcription initiation complex of *Streptomyces coelicolor* shows that the NTPase domain of Fzm_SARP occupies a spatial gap between the BTAD and RNA polymerase in the cryo-EM structure (Figure 5B). This supports the hypothesis that NTPase domains facilitate docking [22,31,32]. We propose that the NTPase domain may provide the energy needed for conformational changes or DNA binding, thereby enabling the SARP to modulate transcriptional activation in a more dynamic manner [32]. For example, the NTPase could facilitate ATP hydrolysis, resulting in structural changes that might enhance or impair interactions with specific promoter elements or co-regulators [22]. While the NTPase domain expands regulatory potential, it also increases both protein size and complexity, which could limit its function in simpler or more constrained environments.

The evolutionary expansion of SARPs from small (e.g., RedD) to medium (e.g., Fzm_SARP) sizes, reflects a gradual increase in regulatory complexity driven by the necessity to integrate multiple physiological signals. This evolutionary path may provide valuable insights for future studies on domain acquisition and loss in regulatory proteins across actinobacteria. Understanding these trade-offs is essential for leveraging SARPs as tools in biotechnology and for unravelling the evolutionary drivers behind regulatory complexity in actinobacteria.

## 4. Materials and Methods

### 4.1. Mutant Generation

Conjugation experiments were performed using *E. coli* donor strains WM6026 and WM3780 and R2 agar without sucrose. The process of spore preparation and conjugation followed the protocol described by Zhang et al. [13]. Apramycin selection was employed to isolate mutants with the integrated DNA. Identification of correct mutants was done with PCR screening, using primers flanking the kasO* promoter and over-expression cassettes, with the wild-type genome serving as a negative control. Sanger sequencing subsequently verified the successful integration of the over-expression cassettes.

### 4.2. Cultivation

Wild-type strains and engineered mutants were cultivated on agar plates at 28 °C for 4 days. Three 5 mm diameter agar plugs from these cultures were used to inoculate 50 mL of seed medium in 250 mL Erlenmeyer flasks. These seed cultures were incubated for four days at 28 °C with shaking at 200 rpm. Subsequently, 2.5 mL of homogenized seed culture was transferred to 50 mL of one of the following fermentation media in 250 mL Erlenmeyer flasks. All cultures were fermented at 28 °C for nine days with shaking at 200 rpm (50 mm throw). Following incubation, cultures were freeze-dried. More details on media composition can be found in Tay et al. [14].

### 4.3. Liquid Chromatography—Tandem Mass Spectrometry (LC-MS/MS) Analysis

Metabolite profiling of the extracts was conducted using an Agilent 1290 Infinity UHPLC system (Agilent Technologies Singapore, Singapore) interfaced with an Agilent 6540 accurate-mass quadrupole time-of-flight (QTOF) mass spectrometer (Agilent Technologies Singapore, Singapore). A 5 µL injection volume was applied to a Waters Acquity UPLC BEH C18 column (Waters Pacific Pte Ltd., Singapore, 2.1 × 50 mm, 1.7 µm particle size). Chromatographic separation was achieved under gradient elution using water (solvent A) and acetonitrile (solvent B), each containing 0.1% (*v*/*v*) formic acid at a flow rate of 0.5 mL/min, holding 2% B for 0.5 min, followed by linearly increasing from 2% to 100% B over 6.7 min and maintaining at 100% B for 1.4 min. Mass spectrometric detection was performed in positive electrospray ionization (ESI) mode with simultaneous acquisition of MS and MS/MS spectra. Source conditions were as follows: nitrogen sheath gas at 12 L/min and 325 °C, drying gas at 12 L/min and 350 °C, nebulizer pressure at 50 psi, nozzle voltage at 1.5 kV, and capillary voltage at 4.0 kV. Internal mass calibration was enabled using reference ions at *m*/*z* 121.0509 (purine) and 922.0098 (HP-0921).

### 4.4. Metabolite and Bioactivity Comparisons

Due to the variation in mutant clone numbers [30] and metabolite production between clones [14,24], we standardized our analysis by selecting a single representative clone per modification for each wild-type strain, prioritizing the clone with the highest number of metabolites produced (Table S6) from each modification derived from the same wild-type strain in each fermentation medium. The representative clones selected for the analyses presented in Figure 2 can be found in Table S4. Data on the 18 wild-type strains and their RedD mutants presented in Figure 2, Figure 3 and Figure 4 were taken from Tay et al., 2024 [14,30] For the metabolite activation case study on surfactins and nocardamines in Figure 3, all available data were considered (i.e., all 774 fermentation extracts) instead of representative clones. New metabolites due to RedD or Fzm_SARP effects were only considered new if they were not observed in the corresponding wild-type strain in any media.

### 4.5. Molecular Networking Analysis

Raw liquid chromatography–tandem mass spectrometry (LC-MS/MS) data were initially processed using MSConvert v3.0.22198-0867718 from ProteoWizard to generate open-source Mascot Generic Format (.mgf) files. To reduce background noise, tandem mass spectrometry (MS/MS) signals with intensity values below 1000 were removed. Classical molecular networking was performed using the online workflow available on the Global Natural Products Social Molecular Networking (GNPS) platform (http://gnps.ucsd.edu). To eliminate residual precursor ions, all peaks within ±17 Da of the precursor ion mass were excluded. Additionally, only the six most intense peaks within a ±50 Da window were retained. The precursor ion mass tolerance and MS/MS fragment ion tolerance were both set to 0.02 Da. A molecular network was constructed, where edges were retained if they exhibited a cosine similarity score above 0.7 with at least six matched peaks. Furthermore, an edge between two nodes was retained only if each node appeared in the other’s top 10 most similar nodes. No upper limit was imposed on the size of molecular families. Spectra within the network were subsequently searched against the GNPS spectral libraries, with the library spectra subjected to the same filtering criteria as the input data. Only matches with a score above 0.7 and a minimum of six matched peaks were retained.

### 4.6. Comparison of Metabolite Production Fold Change Between Wild-Type and Mutant Strains

Raw liquid chromatography-tandem mass spectrometry (LC-MS/MS) data were processed using MSConvert v3.0.22198-0867718 from ProteoWizard to generate Mascot Generic Format (.mgf) files. To reduce background noise, tandem mass spectrometry (MS/MS) signals with intensity values below 1000 were removed. Metabolite yields for each fermentation extract were determined from the processed .mgf files by referencing the MS abundances of all MS/MS spectra with a precursor mass within 0.02 Da and a retention time within 0.4 min of the mean values of the unique metabolites identified using the Global Natural Products Social Molecular Networking (GNPS) [28] online workflow (http://gnps.ucsd.edu). Of the 666 metabolites identified by GNPS as present in both wild-type and mutant (activated) strains, 650 metabolites were successfully quantified using this approach (Table S7). The representative clones selected for the fold change analyses presented in Figure 2 can be found in Table S4.

### 4.7. Biological Assays

Crude extracts were tested for antimicrobial activity against a range of bacterial and fungal strains, including *Klebsiella aerogenes* (EA, ATCC^®^ 13048™), *Pseudomonas aeruginosa* (PA, ATCC^®^ 9027™), *Staphylococcus aureus* (SA, ATCC^®^ 25923™), *Acinetobacter baumannii* (ACB, ATCC^®^ 19606™), and *Aspergillus fumigatus* (AF, ATCC^®^ 46645™). In parallel, cytotoxicity profiling was conducted against human A549 lung carcinoma cells (ATCC^®^ CCL-185™). All assays were conducted in duplicate at a fixed concentration of 100 µg/mL to assess the percentage of growth inhibition compared to a control.

### 4.8. Binding Motif Prediction

Putative SARP-binding motifs within the Fzm biosynthetic gene cluster (GCF_000717915.1) were identified through in silico motif mining. In addition to motif mining, predicted protein–DNA interaction sites were evaluated using Boltz-2 NIM analysis [33]. For this purpose, the N-terminal DNA-binding domains of SARP and RedD were modelled in complex with their respective candidate motifs (Figure S1). Only motifs with interactions comparable to those of AsfR (PDB: 8K60, [22]) in its DNA-bound conformation were included in the comparison of the binding sites (Figure 5).

### 4.9. Structure Modeling and Alignment

Predicted three-dimensional structures of RedD and Fzm_SARP were generated using the ESM protein structure prediction model [34]. The resulting models were aligned and visualized using PyMOL^TM^ v3.1.1 (Schrödinger, LLC, New York, NY, USA). For structural comparison, the recently resolved cryo-EM structure of the large SARP AfsR in complex with the *Streptomyces coelicolor* transcription initiation complex (PDB ID: 8K60, [22]) was used as a reference.

## 5. Conclusions

In this study, we employed a rapid actinobacterial integration pipeline to over-express activators that enhance the production of secondary metabolites. This approach enabled us, for the first time, to characterize a novel medium-sized SARP, Fzm_SARP, in comparison to the well-established and widely used prototypical small SARP, RedD, across 18 actinobacterial strains. Notably, we found that despite Fzm_SARP’s larger size, similarly to RedD, it can effectively induce up-regulation of metabolites and scaffolds across various actinobacterial species. While Fzm_SARP exhibits diversity in the upregulated metabolites, the substantial overlap in upregulated scaffolds indicates that RedD and Fzm_SARP likely target similar pathways. Such insights are essential for understanding the broader implications of SARP families in biochemical pathways. In summary, this compilation of findings has enriched our understanding of the SARP families, providing valuable insights that could inspire further research and application in microbial biotechnology and secondary metabolites production.

## Figures and Tables

**Table 1:** Comparison of small and medium SARPs. RedD and Fzm_SARP are used in this study.

	DIAMOND-BLASTP Outputs from NPDC Database ^a^						
ID	Top MiBIG Hit	BGCs (GCFs)	Main PKS Class (%)	Genomes (Mash Clusters ^b^)	Main Species	Sequence Identity (%) to RedD	Sequence Identity (%) to Fzm_SARP
Fzm_SARP (WP_078621770.1)	Fosfazinomycin A	251 (36)	T1PKS (31%)	246 (52)	*Streptomyces* *Kitasatospora*	33.6	100
BafG [19]	Bafilomycin B1	768 (132)	T1PKS (18%)	553 (126)	*Streptomyces* *Kitasatospora*	30.65	40.61
FdmR [25]	Fredericamycin A	1043 (168)	T2PKS/butyrolactone (10%)	870 (199)	*Streptomyces* *Kitasatospora*	36.12	34.78
RedD [20]	Undecylprodigiosin	89 (5)	NRPS-like/T1PKS (84%)	89 (8)	*Streptomyces*	100	33.6
PapR2 [20]	Fogacin A	878 (122)	T2PKS/butyrolactone (10%)	794 (175)	*Streptomyces*	41.96	36.8

^a^ DIAMOND-BLASTP with e-value, %identity, and query coverage cutoffs of 1 × 10^−10^, 40%, and 80%, respectively within 16,272 genomes from Natural Products Discovery Center (NPDC) database [24], accessed January 2025. ^b^ Mash clusters are used to cluster whole genomes [26].

## Data Availability

The original contributions presented in this study are included in the article/Supplementary Materials. Further inquiries can be directed to the corresponding authors.

## Supplementary Materials

The following supporting information can be downloaded at: https://www.mdpi.com/article/10.3390/ijms262311723/s1.

## References

[1] Atanasov A.G., Zotchev S.B., Dirsch V.M., Supuran C.T., International Natural Product Sciences Taskforce (2021). Natural products in drug discovery: Advances and opportunities. Nat. Rev. Drug Discov..

[2] Shen B. (2015). A New Golden Age of Natural Products Drug Discovery. Cell.

[3] Horbal L., Marques F., Nadmid S., Mendes M.V., Luzhetskyy A. (2018). Secondary metabolites overproduction through transcriptional gene cluster refactoring. Metab. Eng..

[4] He F., Liu X., Tang M., Wang H., Wu Y., Liang S. (2024). CRISETR: An efficient technology for multiplexed refactoring of biosynthetic gene clusters. Nucleic Acids Res..

[5] Ke J., Yoshikuni Y. (2020). Multi-chassis engineering for heterologous production of microbial natural products. Curr. Opin. Biotechnol..

[6] Jiang W., Zhu T.F. (2016). Targeted isolation and cloning of 100-kb microbial genomic sequences by Cas9-assisted targeting of chromosome segments. Nat. Protoc..

[7] Li L., Maclntyre L.W., Brady S.F. (2021). Refactoring biosynthetic gene clusters for heterologous production of microbial natural products. Curr. Opin. Biotechnol..

[8] Gu B., Kim D.G., Kim D.K., Kim M., Kim H.U., Oh M.K. (2023). Heterologous overproduction of oviedomycin by refactoring biosynthetic gene cluster and metabolic engineering of host strain Streptomyces coelicolor. Microb. Cell Factories.

[9] Zhang Y., Feng L., Hemu X., Tan N.H., Wang Z. (2024). OSMAC Strategy: A promising way to explore microbial cyclic peptides. Eur. J. Med. Chem..

[10] Lee Y., Choe D., Palsson B.O., Cho B. (2024). Machine-Learning Analysis of *Streptomyces coelicolor* Transcriptomes Reveals a Transcription Regulatory Network Encompassing Biosynthetic Gene Clusters. Adv. Sci..

[11] Jönsson M., Sigrist R., Petrov M.S., Marcussen N., Gren T., Palsson B.O., Yang L., Özdemir E. (2025). Machine Learning Uncovers the Transcriptional Regulatory Network for the Production Host *Streptomyces albidoflavus*. Cell Rep..

[12] Lim Y.H., Wong F.T., Yeo W.L., Ching K.C., Lim Y.W., Heng E., Chen S., Tsai D., Lauderdale T., Shia K. (2018). Auroramycin: A Potent Antibiotic from Streptomyces roseosporus by CRISPR-Cas9 Activation. ChemBioChem.

[13] Zhang M.M., Wong F.T., Wang Y., Luo S., Lim Y.H., Heng E., Yeo W.L., Cobb R.E., Enghiad B., Ang E.L. (2017). CRISPR–Cas9 strategy for activation of silent Streptomyces biosynthetic gene clusters. Nat. Chem. Biol..

[14] Tay D.W.P., Tan L.L., Heng E., Zulkarnain N., Ching K.C., Wibowo M., Chin E.J., Tan Z.Y.Q., Leong C.Y., Ng V.W.P. (2024). Exploring a general multi-pronged activation strategy for natural product discovery in Actinomycetes. Commun. Biol..

[15] Liu M., Grkovic T., Liu X., Han J., Zhang L., Quinn R.J. (2017). A systems approach using OSMAC, Log P and NMR fingerprinting: An approach to novelty. Synth. Syst. Biotechnol..

[16] Romano S., Jackson S.A., Patry S., Dobson A.D.W. (2018). Extending the “one strain many compounds” (OSMAC) principle to marine microorganisms. Mar. Drugs.

[17] Schwarz J., Hubmann G., Rosenthal K., Lütz S. (2021). Triaging of culture conditions for enhanced secondary metabolite diversity from different bacteria. Biomolecules.

[18] Hwang S., Lee N., Choe D., Lee Y., Kim W., Kim J.H., Kim G., Kim H., Ahn N.-H., Lee B.-H. (2022). System-Level Analysis of Transcriptional and Translational Regulatory Elements in Streptomyces griseus. Front. Bioeng. Biotechnol..

[19] Yan Y., Xia H. (2024). The roles of SARP family regulators involved in secondary metabolism in Streptomyces. Front. Microbiol..

[20] Krause J., Handayani I., Blin K., Kulik A., Mast Y. (2020). Disclosing the Potential of the SARP-Type Regulator PapR2 for the Activation of Antibiotic Gene Clusters in Streptomycetes. Front. Microbiol..

[21] Liu G., Chater K.F., Chandra G., Niu G., Tan H. (2013). Molecular Regulation of Antibiotic Biosynthesis in Streptomyces. Microbiol. Mol. Biol. Rev..

[22] Shi J., Ye Z., Feng Z., Wen A., Wang L., Zhang Z., Xu L., Song Q., Wang F., Liu T. (2024). Structural insights into transcription activation of the Streptomyces antibiotic regulatory protein, AfsR. iScience.

[23] Gao J., Ju K.S., Yu X., Velasquez J.E., Mukherjee S., Lee J., Zhao C., Evans B.S., Doroghazi J.R., Metcalf W.W. (2014). Use of a phosphonate methyltransferase in the identification of the fosfazinomycin biosynthetic gene cluster. Angew. Chem. Int. Ed..

[24] Shen B. (2024). NPDC Portal. https://npdc.rc.ufl.edu/home.

[25] Chen Y., Wendt-Pienkowski E., Shen B. (2008). Identification and utility of FdmR1 as a Streptomyces antibiotic regulatory protein activator for fredericamycin production in Streptomyces griseus ATCC 49344 and heterologous hosts. J. Bacteriol..

[26] Ondov B.D., Treangen T.J., Melsted P., Mallonee A.B., Bergman N.H., Koren S., Phillippy A.M. (2016). Mash: Fast genome and metagenome distance estimation using MinHash. Genome Biol..

[27] Ng S.B., Kanagasundaram Y., Fan H., Arumugam P., Eisenhaber B., Eisenhaber F. (2018). The 160K Natural Organism Library, a unique resource for natural products research. Nat. Biotechnol..

[28] Wang M., Carver J.J., Phelan V.V., Sanchez L.M., Garg N., Peng Y., Nguyen D.D., Watrous J., Kapono C.A., Luzzatto-Knaan T. (2016). Sharing and community curation of mass spectrometry data with Global Natural Products Social Molecular Networking. Nat. Biotechnol..

[29] Tay D., Tan L.L., Heng E. (2022). Training old dogs to do new tricks: A general multi-pronged activation approach for natural product discovery in Actinomycetes. Res. Sq..

[30] Tay D.W.P., Tan L.L., Heng E., Zulkarnain N., Chin E.J., Tan Z.Y.Q., Leong C.Y., Ng V.W.P., Yang L.K., Seow D.C.S. (2024). Tandem mass spectral metabolic profiling of 54 actinobacterial strains and their 459 mutants. Sci. Data.

[31] Wang Y., Yang X., Yu F., Deng Z., Lin S., Zheng J. (2024). Structural and functional characterization of AfsR, an SARP family transcriptional activator of antibiotic biosynthesis in Streptomyces. PLoS Biol..

[32] Leipe D.D., Koonin E.V., Aravind L. (2004). STAND, a class of P-loop NTPases including animal and plant regulators of programmed cell death: Multiple, complex domain architectures, unusual phyletic patterns, and evolution by horizontal gene transfer. J. Mol. Biol..

[33] Boltz-2 Model by MIT. https://build.nvidia.com/mit/boltz2.

[34] Lin Z., Akin H., Rao R., Hie B., Zhu Z., Lu W., Smetanin N., Verkuil R., Kabeli O., Shmueli Y. (2023). Evolutionary-scale prediction of atomic-level protein structure with a language model. Science.

