# Supplementary material for: Enhancing Secondary Metabolite Production in Actinobacteria Through Over-Expression of a Medium-Sized SARP Regulator
Source: Int J Mol Sci. 2025 Dec 3;26(23):11723. doi: 10.3390/ijms262311723 (PMC12692060; doi:10.3390/ijms262311723)
Supplement: Supplementary file 1 [file ijms-26-11723-s001.zip › SI_Figure_v20251120.pdf]

Supplementary Figure

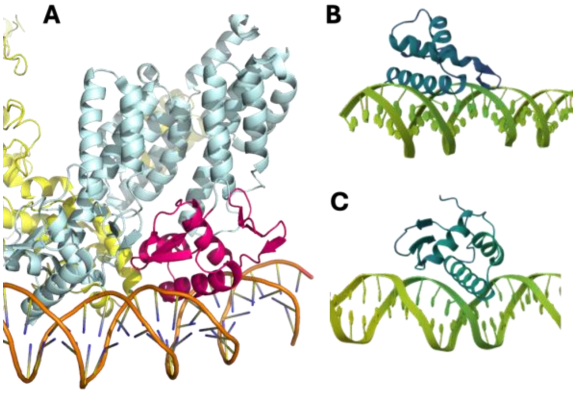

Figure S1. (A) DNA-protein interaction with 8K60 (Domains highlighted in pink) (B) Predicted DNA-protein interaction of RedD with its putative binding motif (Figure 5) (C) Predicted DNA-protein interaction of Fzm_SARP with its putative binding motif (Figure 5).
